# Pivot Teeth

**Published:** 1860-06

**Authors:** A. Berry


### PIVOT TEETH.

BY A. BERRY, D. D. S.

Dr. Koecker, I think, condemned the insertion of pivot teeth. I heard a dentist, in his chair as anatomical professor in a dental college, say, that to set a crown on the fang of a tooth in the mouth was contrary to correct physiology.

Twenty years ago there were many pivot teeth inserted, but now whether this operation should be performed at all is a question for grave discussion at meetings of dental societies.

When pivot teeth are placed on fangs in tolerable healthy condition, of which the operator has the pleasure of destroying the nerves, alveolar abscess will probably not occur for years, but if destitute of vitality, inflammation, &c., may follow forthwith.

It is not improbable that teeth are often removed, for the insertion of others on plates, when they could be pivoted with greater advantage to the patient, as better preserving the contour of the face, and being less troublesome so long as they do well. To be sure extracted teeth never ache, and replacing them on plate pays as well as by pivot, without risk of pain from the operation.

It may sometimes be proper to place artificial crowns on "*dead roots*," and I give the history of three such cases that occurred before the palmy days of small atmospheric plates.

Mr. H. æt. about 23, in good health, with all the superior incisors much decayed, their nerves dead, and the gums around them considerably diseased. The patient was prepared by telling him that trouble would probably follow pivoting his teeth, but that it was best as it would keep off plate teeth a long time. The gums were treated to improve their condition, and the four crowns firmly set a few weeks after. Severe inflammation followed in two or three days, and the patient was kept from his usual business a few days; but now after a lapse of, I think, ten years, he is wearing all the teeth, and with very little trouble since a few weeks after their insertion.

Mrs. C. with central incisors destitute of vitality and crowns gone, with no teeth that would answer well to which clasps could be attached. Prepared by assuring her that if the teeth were inserted on the fangs severe inflammation would probably follow, &c. The teeth were pivoted and she remained at home from her school the next day with an enlargement of the face; but when last heard from, three months ago, she was wearing the teeth comfortably, as she had for about eleven years, the pivots having been renewed once or twice during the time.

Mr. Mc., with the teeth and gums generally much diseased, the superior incisors with most of their nerves dead and crowns nearly gone, but with no teeth proper for sustaining others by clasps. The gums were treated, and the four incisors inserted by pivoting. Severe inflammation supervened directly; but some months since, nine or ten years after the operation, he was wearing them all.

True, this was heroic practice, such as might not add to the reputation of a young operator, but which, performed with proper discrimination, will generally prove satisfactory to the patients.

